# The transition from restrictive anorexia nervosa to binging and purging: a systematic review and meta-analysis

**DOI:** 10.1007/s40519-021-01226-0

**Published:** 2021-06-05

**Authors:** Riccardo Serra, Chiara Di Nicolantonio, Riccardo Di Febo, Franco De Crescenzo, Johan Vanderlinden, Elske Vrieze, Ronny Bruffaerts, Camillo Loriedo, Massimo Pasquini, Lorenzo Tarsitani

**Affiliations:** 1 Department of Human Neurosciences, Sapienza University of Rome, Viale dell’Università, 30, 00185 Rome, RM Italy; 2 Research Group Psychiatry, Department of Neurosciences, KU Leuven University, UZ Gasthuisberg Campus, Herestraat 49, 3000 Leuven, Belgium; 3 Pediatric University Hospital-Department (DPUO), Ospedale Pediatrico Bambino Gesù, Piazza di Sant’Onofrio, 4, 00165 Rome, Italy; 4 Department of Psychiatry, University of Oxford, Warneford Lane, Headington, Oxford, OX3 7JX UK; 5 Department of Neurosciences, Public Health Psychiatry, KULeuven, Leuven RM, Belgium

**Keywords:** Cross-over, Anorexia nervosa, Restriction, Binging and purging, Risk factors, Meta-analysis

## Abstract

**Abstract:**

Numerous studies addressed the topic of behavioral and symptomatic changes in eating disorders. Rates of transition vary widely across studies, ranging from 0 to 70.8%, depending on the diagnoses taken into account and the study design. Evidence shows that the specific transition from restrictive-type anorexia nervosa (AN-R) to disorders involving binging and purging behaviors (BPB) is related to a worsening of the clinical picture and worse long-term outcomes. The aim of this systematic review and meta-analysis is to focus on this specific transition, review existing literature, and summarize related risk factors. Medline and PsycINFO databases were searched, including prospective and retrospective studies on individuals with AN-R. The primary outcome considered was the rate of onset of BPB. Twelve studies (*N* = 725 patients) were included in the qualitative and quantitative analysis. A total of 41.84% (95% CI 33.58–50.11) of patients with AN-R manifested BPB at some point during follow-up. Risk factors for the onset of BPB included potentially treatable and untreatable factors such as the family environment, unipolar depression and higher premorbid BMI. These findings highlight that patients with AN-R frequently transition to BPB over time, with a worsening of the clinical picture. Existing studies in this field are still insufficient and heterogeneous, and further research is needed. Mental health professionals should be aware of the frequent onset of BPB in AN-R and its risk factors and take this information into account in the treatment of AN-R.

**Level of evidence:**

Evidence obtained from a systematic review and meta-analysis, Level I.

## Introduction

Drifting of behavioral and psychopathological patterns can lead to permanent or temporary switches in eating disorder (ED) diagnoses over time [1–3]. While ED diagnoses are useful for describing and managing a patient’s current condition, frequent diagnostic transitions could limit their validity and utility in clinical and research settings. Reported rates of transition between ED diagnoses vary widely across studies, ranging from 0 to 70.8% [4, 5]. The reasons for this high variability are still unclear. Inclusion of patients with different clinical and socio-demographic characteristics and from different settings (e.g., in-patients or out-patients services) could contribute to the variance. Another factor could be the inclusion of patients with anorexia nervosa (AN) without a sub-sample analysis of patients with restrictive-type anorexia nervosa (AN-R) and binge–purge subtype anorexia nervosa (AN-BP), potentially leading to inaccurate estimates. The changing of diagnostic criteria over time (e.g., amenorrhea criterion for AN was removed in DSM-5) might also be a confounding factor. All things considered, existing literature is heterogeneous and does not allow for clear-cut conclusions to be drawn on the subject of diagnostic transition in EDs. Although some research has been conducted, this subject still needs further investigation.

The transition from AN-R to cases involving binging and purging behaviors (BPB) offers an interesting perspective on the subject of diagnostic transition in ED. This specific transition has been shown to entail a worse clinical picture with a longer duration of illness and worse outcomes [6–8]. In line with this, when compared to AN-R, cases involving BPB are associated with a higher prevalence of past traumatic experience [9], comorbid mental disorders [10, 11], maladaptive personality traits [12], suicidality [10], as well as higher levels of somatic and dissociative symptoms (i.e., an interruption in consciousness, identity, environmental awareness, or memory—which is normally well integrated in a healthy person) [1, 13]. From a purely behavioral point of view, AN-R is characterized by a focus on weight loss (accomplished primarily through dieting, fasting and/or excessive exercise) while AN-BP, bulimia nervosa (BN) and binge–eating disorder (BED) are characterized by recurrent episodes of binging (eating large amounts of food in relatively short time while experiencing a sense of loss of control). Furthermore, in AN-BP and BN, binging behaviors are usually followed by a variety of harmful behaviors aimed at regaining control of weight such as vomiting, the use of laxatives and diuretics or excessive physical activity (purging behaviors) [1]. Interestingly, although the behavioral difference is pronounced, no significant differences were found when objectively measuring behavioral impulsivity in patients with different ED diagnoses [14]. Furthermore, some studies focused specifically on patients with AN-R transitioning to BPB (e.g., [8]) and it has been reported that 36% of patients with AN-R develop BN over time [15].

Relevantly, risk factors for transitioning between EDs have been highlighted. Physiological and environmental factors, such as higher premorbid body mass index (BMI) [4] and conflictual family environment [15], as well as depression, substance abuse, panic disorder and obsessive–compulsive disorder [2, 3], have been associated with transitions between ED diagnoses. However, scarcity and heterogeneity of available evidence might prevent this clinically notable information from reaching mental health professionals. Accessibility to an organic source of knowledge on these data could be of clinical relevance, especially in the management of first onsets and younger patients.

This systematic review and meta-analyses aims to compare contents and provide clearer figures of available data on the transition from AN-R to BPB. It also aims to provide a unified, structured source of information on risk factors for this specific transition. This could hopefully foster further studies on the topic of diagnostic transition in EDs and ultimately push toward effective prevention strategies and specific early interventions for avoiding chronicization in the treatment of AN-R. In pursuit of its aims, this review tried to answer the following research questions: “what is the number of patients with AN-R who undergo an onset of BPB?” and “is it possible to predict which patients are at high risk for a transition from AN-R to BPB?”.

## Materials and methods

### Data sources

We developed our review and meta-analysis according to the PRISMA statement [16]. The primary search strategy involved exploring databases (Medline, PsycINFO) through December 2020, to identify relevant, peer-reviewed, articles in English on long-term outcomes of patients with AN-R. The final search syntax was the following: (anorexia nervosa) AND ((follow-up study) or (course) or (predictors) or (evolution) or (crossover) or (transition) or (prediction)) AND ((bulimia) or (vomiting) or (binging) or (purging)). A filter for English language was applied. Gray literature was searched using multiple resources such as Scopus and reference tracing.

### Eligibility criteria

Selection of the evidence was structured in three progressively more selective phases:

Phase 1—Title screening including only studies on the long-term outcome of patients who received the diagnosis of AN at admission.

Phase 2—Abstract screening applied all criteria of phase 1 and, in addition, only included studies on patients with a specific diagnose of AN-R.

Phase 3—Full-text screening applied all criteria of phase 2 and included only studies reporting data on the transition from AN-R to BPB that used validated diagnostic instruments.

Two authors (Serra & Di Nicolantonio) independently reviewed titles and abstracts of retrieved references. Then, the same two researchers independently reviewed the full-text versions of the articles to confirm their eligibility for inclusion. The age of participants, study setting, design and sample size were not criteria for exclusion. All disagreements were resolved in a consensus meeting with the team. Each included study was assessed using the Newcastle Ottawa Scale, an instrument developed to assess the quality of non-randomized studies on three broad perspectives: the selection of the study groups; the comparability of the groups; and the ascertainment of either the exposure or outcome of interest for case–control or cohort studies, respectively [17].

### Data extraction

Two authors (Serra & Di Nicolantonio) independently extracted data from each of the included references: *authors’ names*, *year of publication*, *sample size*, *drop-out rate*, *diagnostic criteria*, *mean BMI*, *rates of BPB onset*, *outcome criteria*, *therapeutic regimen* (in-/out-patients/other), *assessment tools*, *risk factors*, *mean age*, *mean age at onset* and *duration of illness*, *duration of follow-up*, *year* and *country* of the study. Primary outcome was the rate of onset of BPB during follow-up for a AN-R diagnosis (i.e., the proportion of participants that engaged in BPB at some point during follow-up).

### Statistical analysis

Analyses were performed using the “metaprop” command of STATA 16. We calculated pooled incidence of BPB onset with its 95% confidence interval (CI) using a random effects meta-analytic model. We also quantified heterogeneity using the I-squared measure.

## Results

**Fig. 1:**
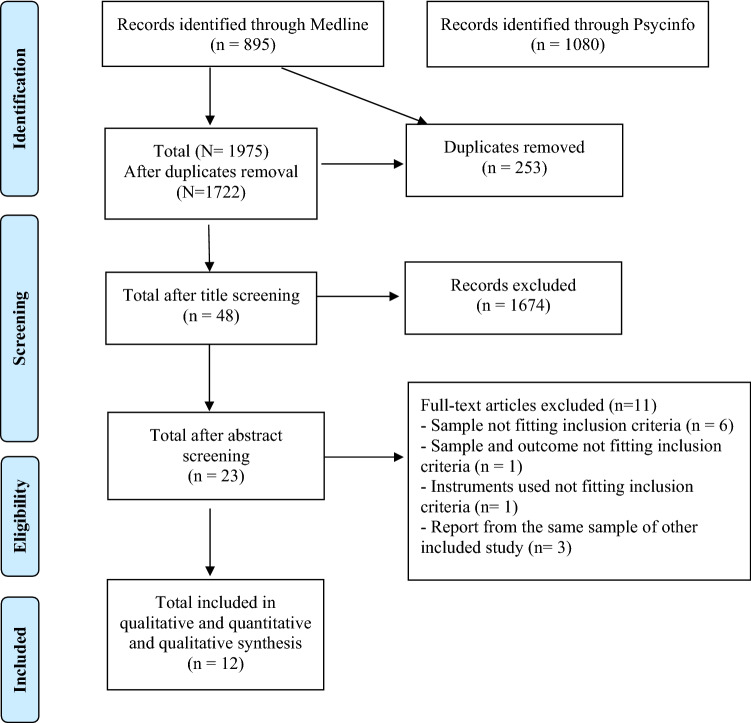
Flowchart of the screening and selection of literature included in qualitative and quantitative analysis

A total of 1975 records were identified through database search, with 1722 remaining after duplicate removal. The screening of titles and abstracts led to 23 studies. The full-text evaluation led to the exclusion of 11 studies (Fig. 1). Reasons for non-inclusion in the review were diagnostic instruments not fitting inclusion criteria (*n* = 1), report from the same sample of another included study (*n* = 3), sample not fitting inclusion criteria (*n* = 6) and sample and outcome not fitting inclusion criteria (*n* = 1). The twelve studies included accounted for a total of 725 patients with a baseline diagnosis of AN-R. Table 1 summarizes results and characteristics of the included studies. All but three of the retrospective studies had a prospective design, [15, 18, 19]. The mean study sample size consisted of 60.41 patients (SD = 24.25) with only three studies having a sample size lower than 50 [4, 20, 21] and only one with less than 30 [22]. All patients were women and the mean age at baseline was 22.02 years (SD = 5.08). Reports were gathered from many different areas of the globe, improving generalizability of the findings: a total of five studies recruited patients from the USA [8, 10, 15, 20, 22], three from Italy [2, 11, 18], while the remaining four studies were respectively from Germany [21], Japan [19], Sweden [4] and the UK [23]. Only two studies enrolled adolescents [4, 8]. As shown in Table 1, six studies used DSM-IV diagnostic criteria [10, 11, 15, 18, 19, 21], three studies used DSM-III [8, 20, 22], one study used both DSM-III and DSM-IV [4], and one used ICD-10 [23]. Three studies enrolled in-patients [8, 20, 21], three enrolled out-patients [2, 11, 19] and two studies enrolled patients from mixed settings [4, 23]. Four studies did not report the specific setting of recruitment [10, 15, 18, 22]. Outcome criteria used were the onset of BN [15, 18, 22], the onset of BPB with a specified frequency [10, 11, 23], the onset of BPB with no frequency cut-off [2, 4, 20], the onset of any BPB disorder [19, 21], and the onset of objective binge-eating only (defined as having eaten more than other people would consider normal, as opposed to the subjective sensation of having eaten excessively) [8]. The mean age at onset of the AN-R was assessed in eight studies and was 17.44 years (SD = 2.52); mean duration of illness was 5.12 years (SD = 3.59). Average baseline BMI was 16.63 kg/m^2^ (SD = 2.59). The follow-up duration of studies was long with an average of 9.05 years (SD = 6.59), ranging from a minimum of one up to 20 years. In two studies, patients died during follow-up [10, 20], with a mean mortality rate of 2.5%. The NOS evaluation revealed good quality level and the absence of substantial source of bias in the selected studies (data available upon request).

**Table 1:** Characteristics of the studies included in the review meta-analysis

	*N*	BPB onset rate, %	Follow-up time, y	Age, y (SD)	Sample	Considered outcome	Risk factors (reported effects)	BMI at T0	Remission rate %	Diagnostic criteria	Country	Recruitment year
Anderluh et al. [23]	55	38	20	28.4 (10.50)	In- and out-patients	BPB once in 2 weeks for 3 months	NR	16.4 kg/m^2^	4.70%	ICD-10	UK	NR
Castellini et al. [2]	76	43.4	6	27.2 (9.10)	Out-patients	Any BPB	Unipolar depression	16.5 kg/m^2^	52.10%	DSM-IV excluding amenorrhea	Italy	2003
Eckert et al. [20]	40	53.5	8	20.0 (5.20)	In-patients	Any BPB	NR	31.1% below IBW	23.70%	DSM-III-R	USA	1977
Eddy et al. [10]	51	62.2	8	22.4 (NR)	Treatment-seeking	Any BPB once weekly or more for 8 weeks	NR	25% below IBW	33.30%	DSM-IV	USA	1987
Fichter et al. [21]	30	30.0	2	24.9 (6.70)	In-patients	Diagnoses of DSM-4 AN-BP or BN	NR	14.3 kg/m^2^	60%	DSM-IV	Germany	1985
Lantz et al. [4]	48	70.8	18	14.26 (1.62)	Students	Any BPB	Highest premorbid BMI	5.5th percentile	NR	DSM-III, DSM-IV	Sweden	1985
Monteleone et al. [18]	70	34.2	Retrospective	25.89 (NR)	Treatment-seeking	BN	Novelty seeking Self-directedness Age at onset Duration of the illness Maximum past BMI	21.8 kg/m^2^	NR	DSM-IV	Italy	2011
Nishimura et al. [19]	80	45.0	Retrospective	24.00 (7.43)	Out-patients	Diagnoses of DSM-IV AN-BP or BN	Duration of illness Maximum lifetime BMI BDI score Parental criticism Self-directedness	15.27 kg/m^2^	NR	DSM-IV	Japan	NR
Smith et al. [22]	16	37.5	6	15.5 (NR)	Treatment-seeking	BN	NR	15.47 kg/m^2^	NR	DSM-III-R	USA	1987
Strober et al. [8]	77	29.0	12,5	14.40 (0.83)	In-patients	Objective binge eating only	Hostile attitudes toward family Lack of parental expressed empathy/affection toward patient	14.1 kg/m^2^	75.80%	DSM-III-R	USA	1980
Tenconi et al. [11]	94	22.3	1	20.60 (4.06)	Out-patients	At least 4 episodes of binging in 2 weeks	Early menarche Body dissatisfaction	15.9 kg/m^2^	NR	DSM-IV	Italy	1993
Tozzi et al. [15]	88	36.36	Retrospective	26.75 (8.99)	Mixed cohort	BN	Self-directedness Parental criticism	20 kg/m^2^	NR	DSM-IV	USA; Canada	2003

*BPB* binging/purging behaviors, *NR* not reported, *AN-BP* anorexia nervosa binging/purging type, *BN* bulimia nervosa, *SD* standard deviation, *BMI* Body Mass Index, *NR* not reported

The pooled rate of AN-R patients who underwent an onset of BPB was 41.84% (95%CI 33.58–50.11). The pooled remission rate was 41.91% (95%CI 15.96–67.85; *I*^2^ = 97.23%).

A total of seven of the studies identified risk factors for the onset of BPB in AN-R (Table 1) [2, 4, 8, 11, 15, 18, 19]. Two studies presented results from bi-variate models [18, 19]. All other studies reported results from multivariate models and are presented in Table 2, according to their potential treatability (modifiable/unmodifiable).

**Table 2:** Synopsis of risk factors and relative available data on significance and effect size

Unmodifiable	**Age at menarche** (B = − 0.26, Wald = 3.70, *p* = 0.054, Exp(B) = 0.77) **Highest premorbid BMI** (*κ*^2^(2) = 12.40, *p* = 0.002, *R*^2^_Snell_ = 5.23)
Partially modifiable	**Lack of parental expressed empathy or affection toward the patient** (OR = 3.1, 95% CI 1.1–8.6, *p* = 0.028) **Parental criticism** (*χ*^2^ = 5.00, df = 1, *p* < 0.03, OR = 1.83, 95% CI 1.08–3.12) **Self-directedness** (*χ*^2^ = 7.30, df = 1, *p* = 0.007, OR = 0.45, 95% CI 0.26–0.81)
Modifiable	**Body dissatisfaction** (*B* = 0.11, Wald = 10.14, *p* = 0.002, Exp(B) = 1.11) **Hostile attitude towards the family** (OR = 6.7, 95% CI 2.2–20.2, *p* = 0.0007) **Presence of unipolar depression** (OR = 7.38; 99% CI 1.13–47.96)

Bold values used to highlight the predictors in contrast to the statistical data

Note. Only risk factors identified using multivariate analysis are presented. Variables were assigned to “partially modifiable” if affecting the patients’ past as well as their future development

*BMI* body mass index, *OR* odds ratio

## Discussion

This is the first systematic review and meta-analysis of available evidence on the onset of BPB in AN-R patients. The review also includes a synopsis and categorization of reported risk factors for the onset of BPB. The onset of BPB in AN-R is consistently reported across studies, with a pooled 41.84% of the patients undergoing this transition at some point during follow-up. The pooled remission rate of 41.91% suggests that the vast majority of non-remitting patients will eventually undergo a transition to BPB. It is hard to define a specific, single psychopathological phenomenon underlying cross-over in eating disorders. Many factors are surely involved and, as for the onset of BPB in AN-R patients some of these factors were highlighted in seven of the included studies (Table 1).

Compared to patients with stable AN-R, undergoing an onset of BPB was consistently related to a higher premorbid BMI in well-designed bivariate and multivariate models [2, 4, 15, 18]. Although further research is needed to explain this finding, researchers have hypothesized that a higher premorbid BMI could be the sign of a greater pre-existing appetitive drive or a weaker appetitive inhibition potentially facilitating the onset of binging and/or purging (the latter with the aim of weight control) while on a restrictive diet [4]. This hypothesis is also in line with genetic studies showing how obesity-related genes such as the chromosomal region 10p and the preproghrelin gene single nucleotide polymorphisms are recognized susceptibility factors for the development of bulimia [24, 25]. Higher premorbid BMI could also be related to higher levels of body dissatisfaction, which was also correlated to higher risk of BPB onset in AN-R [11].

Another strong finding across multivariate models is the association between onset of BPB and various aspect of familial relations such as patients’ hostile attitude towards their family, high parental criticism and lack of parental expressed empathy/affection towards the patient [8, 15, 19]. It is important to notice how many studies in the field have shown an association between BPB and the presence of a history of trauma [9, 15, 26] and that, while psychological trauma is associated to diagnostic instability and BPB [27, 28], it is not associated to AN-R [29]. In line with clinical theories such as the theory of “escape from self-awareness” [30] or the theory of “complex relational trauma” [31], given the available evidence, it is possible to state that a tense and conflictual family environment implies a higher risk of BPB onset in AN-R. Interesting in this regard, is the emerging evidence of the role of emotional dysregulation and depression in the relation between past traumatic experiences and EDs [32, 33]. More specifically, emotional dysregulation and depression seem to have a mediating effect-linking trauma and emotional overeating, which is commonly reported by patients with eating disorders involving BPB [33, 34]. This is in line with the presented evidence on unipolar depression, which showed a strong prospective association with the onset of BPB [2]. Further research is needed to understand whether there is a causal relation between unipolar mood disorders and the onset of BPB in AN-R. The presence of unipolar depression could, in fact, be a sign of severity or have a unique role in the onset of BPB in patients with AN-R.

One last factor to keep in mind when studying this phenomenon is starvation itself. In fact, re-nourishing after prolonged food deprivation is associated with binging, food hoarding, depressive mood and other impulsive behaviors, leading to the hypothesis of a causal link between starvation and the development of BPB [35, 36]. As highlighted in the milestone Minnesota semi-starvation experiment and later confirmed in non-experimental conditions, binging and purging behaviors can also emerge during nutritional rehabilitation of individuals constrained to protracted dietary restriction [37–39].

Overall, highlighted predictors for the onset of BPB in AN-R seem to point at a biopsychosocial model involving psychological, familial and metabolic factors. In line with this, we hypothesize that no single cause can lead to such a complex transformation, rather a concausal chain pushing against the single patient’s will and psychological resources.

### Limitations and future research directions

Present results should be interpreted in consideration of several limitations. First, some characteristics limit the generalizability of available data: three studies had a sample size lower than *n* = 50 [20–22]; reports only considered women; and ten of the studies included patients regardless of current age and age of onset in spite of the early onset of AN. Second, the specificity of our research question led to the inclusion of a low number of studies, potentially limiting the generalizability of our findings. However, we believe that the focus on AN-R rather than a general group of patients with AN increases the strength of our findings. The proportion of AN-R patients undergoing an onset of BPB in any given study is related to the specific definition of “transition” and its measurement in a given study, limiting the availability of data fit for a meta-analysis. However, results showed a narrow 95% CI proving adequate homogeneity of included studies. Nevertheless, although the selection was strict it is possible that the samples include non-homogeneous clinical presentations, diagnosable as AN-R, of which slight differences are understandably not reported. Future studies in this field should apply more reliable criteria for the definition of BPB onset. In our opinion, despite seemingly different criteria used to assess the transition to BPB, the onset of any impulsive behavior (i.e., isolated binging, diagnosis of AN-BP, any BPB with a low frequency or others) is the lowest common denominator of a dramatic change in the clinical course of AN-R. Risk factors identified in included studies should be considered in future research examining predictors, mediators and moderators of the onset of BPB in AN-R patients. Given the ego-dystonic nature of binging behaviors, it is possible that sharing with patients the information that fasting may lead to BPB could increase motivation and adherence to nutritional rehabilitation and therapeutic plans. Future research could test this hypothesis to help clinicians in the management of this critical aspect of AN-R therapy.

## Conclusions

Almost half of the patients with a diagnosis of AN-R will eventually undergo an onset of BPB, marking an evolution of their clinical condition with longer duration of illness and reduction of their possibility for a sustained recovery [7, 8, 40]. As highlighted in a recent study [41], general practitioners, nutritionists and mental health professionals have a key role in rapidly directing patients to specialist care settings to provide appropriate care. This is critical in the prevention of the permanent risks associated with starvation, the onset of BPB and the general worse outcomes related to a late diagnosis [6, 42, 43]. Confirmed risk factor for the onset of BPB should be systematically assessed by clinicians in patients AN-R and specific treatments (such as family therapy) should be considered in patients at high risk for the onset of BPB, especially in the evaluation and treatment of first onsets. This could lead to more prompt interventions for preventing chronicization and the evolution of the clinical picture.

### What is already known on this subject?

Rates of diagnostic transition in ED vary widely across studies, ranging from 0 to 70.8%. Heterogeneity of existing research makes it hard to interpretate available data. The specific transition from restrictive-type anorexia nervosa to disorders involving binging and purging behaviors has been related to a worse long-term prognosis. Over the years, some risk factors for this transition have been identified.

### What this study adds?

This study focuses on the literature from a neglected area of research, suggesting that the transition from restrictive-type anorexia nervosa to disorders involving binging and purging behaviors is common. Results are consistent across country of origin, decade when the study was performed, design of the study and clinical/nonclinical settings. Some established risk factors for the transition could be targeted in therapy.

## Data Availability

A database containing data from the different phases of the selection of articles was shared during the submission process (as additional material was not for review). The database will be shared upon request.

## Code availability

Code will be shared upon request.
