# Correction to: The transition from restrictive anorexia nervosa to binging and purging: a systematic review and meta‑analysis

**DOI:** 10.1007/s40519-021-01244-y

**Published:** 2021-07-02

**Authors:** Riccardo Serra, Chiara Di Nicolantonio, Riccardo Di Febo, Franco De Crescenzo, Johan Vanderlinden, Elske Vrieze, Ronny Bruffaerts, Camillo Loriedo, Massimo Pasquini, Lorenzo Tarsitani

**Affiliations:** 1 Department of Human Neurosciences, Sapienza University of Rome, Viale dell’Università, 30, 00185 Rome, RM Italy; 2 Research Group Psychiatry, Department of Neurosciences, KU Leuven University, UZ Gasthuisberg Campus, Herestraat 49, 3000 Leuven, Belgium; 3 Pediatric University Hospital-Department (DPUO), Ospedale Pediatrico Bambino Gesù, Piazza di Sant’Onofrio, 4, 00165 Rome, Italy; 4 Department of Psychiatry, University of Oxford, Warneford Lane, Headington, Oxford, OX3 7JX UK; 5 Department of Neurosciences, Public Health Psychiatry, KULeuven, Leuven RM, Belgium

## Correction to: Eating and Weight Disorders - Studies on Anorexia, Bulimia and Obesity 10.1007/s40519-021-01226-0

Unfortunately, some authors' first name and surname were incorrectly published in the original publication. The complete correct names of authors are given below.

Riccardo Serra, Chiara Di Nicolantonio, Riccardo Di Febo, Franco De Crescenzo, Johan Vanderlinden, Elske Vrieze, Ronny Bruffaerts, Camillo Loriedo, Massimo Pasquini, Lorenzo Tarsitani.

The original article has been corrected.

